# Social Image of Nursing. An Integrative Review about a Yet Unknown Profession

**DOI:** 10.3390/nursrep11020043

**Published:** 2021-06-07

**Authors:** Macarena López-Verdugo, Jose Antonio Ponce-Blandón, Francisco Javier López-Narbona, Rocío Romero-Castillo, María Dolores Guerra-Martín

**Affiliations:** 1 Faculty of Nursing, Physiotherapy and Podiatry, University of Seville, 41009 Seville, Spain; macarena.lv93@gmail.com (M.L.-V.); guema@us.es (M.D.G.-M.); 2 Red Cross Nursing School, University of Seville, 41009 Seville, Spain; fjlona@cruzroja.es (F.J.L.-N.); rocio.romero@cruzroja.es (R.R.-C.)

**Keywords:** nursing, public image, nursing image

## Abstract

Background: Nursing is a discipline on which stereotypes have persisted throughout its history, considering itself a feminine profession and subordinated to the medical figure, without its own field of competence. All this leads to an image of the Nursing Profession that moves away from reality, constituting a real, relevant and high-impact problem that prevents professional expansion, and that has a direct impact on social trust, the allocation of resources and quality of care, as well as wages and professional satisfaction. The aim of this review was to identify and publicize the published material on the social image of Nursing, providing updated information about the different approaches to the subject. Methods: An integrative review of the literature has been made from primary sources of information published from 2010 to 2020. For this, the databases CINAHL, Scopus, SciELO, Dialnet and Cuiden have been consulted. Results: In total, 17 articles have been included in the review, with qualitative, quantitative, and even bibliographic reviews performed in countries such as Spain, Egypt, Argentina, Iran, Venezuela, Turkey, United Kingdom, and Australia. The results of those papers mostly showed that society has misinformation about the functions performed by nursing professionals, which is based on myths and stereotypes. Conclusions: Nursing is a profoundly unknown and invisible profession, as society continues without recognizing its competence, autonomy and independence.

## 1. Introduction

“Care” is the essence of Nursing. This care is conducted by means of various actions aimed at improving or mitigating the discomforts caused by a disease process or to preserve health. For this reason, the individuals who exercise the Nursing Profession must possess the concrete skills, knowledge and intellectual ability that allow them to solve the real or potential problems of the people at which care is targeted, by resorting to critical thinking and effective communication [1]. An emphasis on care of an instrumental nature has been traditionally identified. The perception of patients differs from that of nurses; patients perceive a lower level of personal care than the one nurses believe that they deliver. Caring behaviors are affected by the working environment, nurses’ emotional intelligence and coping skills, and socio-demographic characteristics [2]. In fact, the perceived care in the nurse-patient relationship is high and instrumental in nature, and it can be stated that nurses consider that through their behaviors they transmit more care than the users perceive they receive [3].

Throughout history, Nursing has been influenced by gender considerations, for being understood that care is an activity inherent to women. In this way, its evolution and development has been conditioned, giving rise to a profession that is struggling to attain the goals established. In our times, although in theory we should have overcome the sexist roles, we observe that the profession is still being marginalized in different ways and that there is a devalued image of the activities performed by female and male nurses, although they perform indispensable work [4,5]. In addition, as a consequence of the stereotypes about the gender roles, the male collective in Nursing is still a minority, thus limiting the development of its professionals, which, in turn, has a negative impact on their image [6].

On the other hand, the communication media frequently reflects stereotypes that result in degrading and exerts a negative influence on the image of the Nursing Profession [7]. In this way, it is evident how in films, TV series, commercials, and more serious programs of greater repercussion such the news, a distorted image of the Nursing competences is projected, which has nothing to do with the true commitment of this collective towards the health of the population [8].

Likewise, it can be asserted that the professional identity of Nursing does not coincide with the social image of the profession [9]. However, the first is influenced by the second, and vice versa. Consequently, social image has a negative impact on the construction of professional identity, because the population conditions the thoughts, beliefs and behaviors of female and male nurses [10]; and, in its turn, what the Nursing professionals convey through their actions is going to determine the existing public image of the profession. It is then spoken of as a “weak” identity of the Nursing Profession that has been shaped based on the stereotypes which have persisted throughout its history, so that its professionals are probably going to project an inadequate image of Nursing to the population, with a negative impact on the social conception regarding the Nursing discipline [11]. 

By social image we understand an iconic and simple creation based on stereotypes, which represents through attributes the discourse of a concrete sociocultural setting regarding a social reality. It is a concept agreed upon by the members of a society about the social representations constructed in view of a reality that aims to visually summarize certain social discourse through the characteristics that best fit the ideological or moral content [12]. The public image of Nursing is given by its external aspect, by the mental scheme of the concept of the profession (which is determined by words or images), and to what it resembles when related to other professions. The set of characteristics that are repeated by means of the various mental representations of the individuals is what constitutes the social image of the profession [10].

On the other hand, we can talk about social prestige or status, which is given by the public recognition attributed by the professional field [13], which must be proportional to the social work that is performed. In order to attain greater social recognition, it is necessary that both work and educational institutions show a professionalized image of Nursing, giving visibility to all the care actions provided to the population, showing their scientific knowledge and competence, as well as assertiveness when making decisions and in problem resolution [14]. Therefore, Nursing professionals must be considered a key component within the inter-professional team, for which it is fundamental to construct and reassert the concept of professional identity or, in other words, the conception that the individuals who exercise the profession have of it, and of themselves [15].

It is of vital importance that the Nursing professionals reflect on what they have achieved, and on the situation they are in, to be able to correctly define what they are and what they want to achieve, projecting it to society to give visibility to their essence [16]. 

Now focusing on the image that the population has of Nursing, it is worth noting a study conducted in the United States. Its objective was to know the perception held by the population regarding Nursing. The following results stand out: 17% of the young individuals and 15% of the adults answered that the main function of female and male nurses is to assist the medical figure; just 39% of the young individuals and 50% of the adult population stated that Nursing professionals made use of critical thinking, intelligence and problem resolution; and 40% thought that nurses helped the medical professionals [17].

Therefore, it is not surprising that, in the IV Nursing Conference of the Valencian Community, it was concluded that 60.5% of the patients did not trust in the health advice provided by the Nursing professionals [18]. In addition, the users are under the wrong idea that the Nursing activities are limited to the care scope in health institutions [19] and still perceive the Nursing professionals as subordinates of the medical collective [20]. 

Some of the answers given by patients in a study conducted in Spain to open questions are as follows: “I think that, in order to know how to treat a disease, there are the doctors; nurses are more for social dealings than for their knowledge”, “Regarding the social image, I believe that it’s quite positive, possibly one of the professions that is seen as most kind”, “Then when the nurse is at your bedside you tell her everything, it’s easier to talk”. It is therefore seen that both male and female patients value more kindness and closeness than the nurses’ knowledge [21].

Nowadays, strengthening the image of Nursing supposes a challenge for its professionals. In countries such as Spain, many efforts have been made to manage appropriate tools to evaluate, during the undergraduate training period of future nurses, aspects such as academic satisfaction [22] or the perception of care that clinical instructors demonstrate to students, considering the strong impact it has on their future relationships with patients, relatives, and other health professionals [23]. However, the fact that the social image of Nursing does not coincide with the reality of the profession implies a series of repercussions ranging from high professional dissatisfaction or burnout to diversity of conflicts that reduce potential and work capacity in multidisciplinary-interdisciplinary teams and prevent the expansion and advancement of the profession, without overlooking the fact that this situation directly affects quality of care and users’ satisfaction. In addition, it also has an impact on the social trust deposited in the Nursing professionals, on resource allocation and work overload, as well as on the salaries of its professionals [24]. Consequently, it is a real and relevant problem, as well as one of high impact. By means of this paper, the intention is to contribute to eliminating the stereotypes and to construct a more professional image of the discipline. 

The Nursing Profession has experienced numerous changes throughout history, with both internal and external aspects exerting an influence on its evolution [25]. The scope of Nursing performance has been expanded, so that, in addition to exercising the profession at the assistance level and managing care, nurses are a key component in the industry, education and research, among other aspects, performing their functions independently [26]; but, is society aware of these changes? “Does the population start to understand what we are or what we aspire to be?” [16].

It is fundamental to communicate what Nursing is, what knowledge the Nurses have, and what Nursing provides as a whole to the profession [27], as well as to devise strategies aimed at showing society what the true role of the profession is, correcting false ideas and providing an image of Nursing that adjusts to reality, so that those who exercise the profession control the communication process. giving visibility to what we are and what we want to be [8]. Contemporary health care requires that female nurses know who they are and the role they play, how to identify and update their mission in society, and how to communicate so to others [28].

Given the above, the objective of this paper is to identify and disclose the material published on the social image of Nursing, providing updated information about the different approaches on the topic.

## 2. Materials and Methods

In order to develop this review objective, and applying the PICO (Patients, Interventions, Comparison, Outcomes) model for the formulation of clinical questions in the practice based on evidence [29], the following PICO question was established: “What is the social image that general population/patients have about the role and performance of nurses?”, defining the following elements of the question:

✓ Patients/population: General population or general patients from any cultural context
✓ Intervention: Role or performance of nursing professionals
✓ Comparison: No comparison group (not applicable)
✓ Outcomes: Image that society or patients in general have about these professionals.

To elaborate this integrative review and knowledge update paper, primary information sources have been used, which have been obtained through the CINAHL, Scopus, SciELO, Dialnet and Cuiden databases. Searches were also conducted in PubMed, Medline, PsycInfo and Enfispo, but no articles were found that responded to the objectives of the review. The search was conducted between November and December 2020. The subsequent process for the selection of the articles was exactly the same for all the databases used, using the following descriptors: “Nursing” “Social perception” “Patients” and “Population”, all included in MeSH (Medical Subject Headings) and in DeCS (Descriptores en Ciencias de la Salud, created by BIREME, Latin American and Caribbean Centre of Information in Health Sciences). Later, in order to complement the search, the free descriptors “Nursing Image” and “Clients” were added. The search strategy applied systematically combining the Boolean operators with the aforementioned descriptors was ((“Nursing” AND “Social perception”) AND (“patients” OR “population”)). The search was repeated incorporating the aforementioned descriptor “Nursing Image”, instead of “Nursing” AND “Social perception”, defining the following search strategy: ((“Nursing Image)” AND (“patients” OR “population”)). Finally, the free term “client” was included with the Boolean “OR” together with the rest of the terms mentioned, defining the following strategies:

✓ ((“Nursing” AND “Social perception”) AND (“patients” OR “clients” OR “population”))
✓ ((“Nursing Image”) AND (“patients” OR “clients” OR “population”))

The inclusion criteria were as follows:

✓ Original or review articles showing studies of a quantitative or qualitative nature, including literature reviews, that aim to identify and/or describe the image or possible stereotypes regarding the Nursing Profession.
✓ Studies of any of the described methodologies whose purpose is to describe what nurses do, which addresses the point of view of the general population, whether or not they have had experiences with health care; the perception of health professionals and/or nursing students, as well as the self-image of the Nursing Profession.
✓ Year of publication (2010–2020): The search was prefixed in the last 10 years to focus on the most current image of the profession and to not include studies that may get results that are biased by the more historical vision of the profession.
✓ Language (Spanish or English).
✓ Access to the abstracts and full text.

On the other hand, the exclusion criteria were the following:

✓ Repeated articles
✓ Not having access to the full text
✓ Studies that were not related to the objective.

Two reviewers carried out the search strategy in the selected databases, applying the pre-established inclusion and exclusion criteria separately, consenting to the documents to be included in the review in a second phase.

For the analysis of the contents of the selected articles, the full texts were obtained and following the standardized guidelines for integrative reviews [30,31] were undertaken in duplication and separately, collecting the variables of authorship, study methods, bias risks, intervention and comparison groups (if pertinent), results obtained and the main conclusions. The data extracted simultaneously by the two researchers were later compared and completed between each other, in such a way that, after this joint analysis, articles were discarded in which it was mutually agreed and confirmed that at least one inclusion or exclusion criterion was not met.

Following the PRISMA recommendations [32], the risk of bias of the included studies was assessed by evaluating the methods followed in the articles. Quality appraisal was performed to select only the highest quality papers, in order to obtain the most reliable results and to identify the strengths and weaknesses of the selected articles, that could compromise the validity of the present review results [33]. For the evaluation of the methodological quality of the articles, the assessment tool for Strengthening the Reporting of Observational Studies in Epidemiology (STROBE) was applied [34]. For qualitative studies, Standards for Reporting Qualitative Research (SRQR) [35] was used and finally, in case of Literature Reviews, PRISMA 2020 checklist statement was applied in order to assess the quality of the papers [32].

The purpose of these appraisal tools was to assess the methodological quality of a study and to determine the extent to which a study has addressed the possibility of bias in its design, development and analysis. It consists of the application of different checklists that can be assessed as “yes”, “no”, “unclear” or “not applicable”. The results of this appraisal were used to inform synthesis and interpretation of the results of the studies reviewed. The research team agreed to include articles that obtained one item scoring “no” and one item scoring “unclear” at most. Two studies out of the 19 papers included in the methodological quality appraisal were excluded because they obtained a negative assessment in two items or more. Two researchers assessed the papers independently and agreed on the conclusions by consensus. For the data extraction process, a data extraction form was developed prior to including the variables to be collected. These variables were stated by the research team to answer the research question and meet the aim of this review. The variables were authorship, year of publication, country, aim of the study, study population, study methods, results, outcomes, and conclusions. Data was extracted simultaneously from primary research selected papers by two researchers. The two data extraction forms obtained were compared, and they completed each other. The information obtained was analyzed, and a narrative synthesis was carried out describing the results [36].

## 3. Results

**Figure 1:**
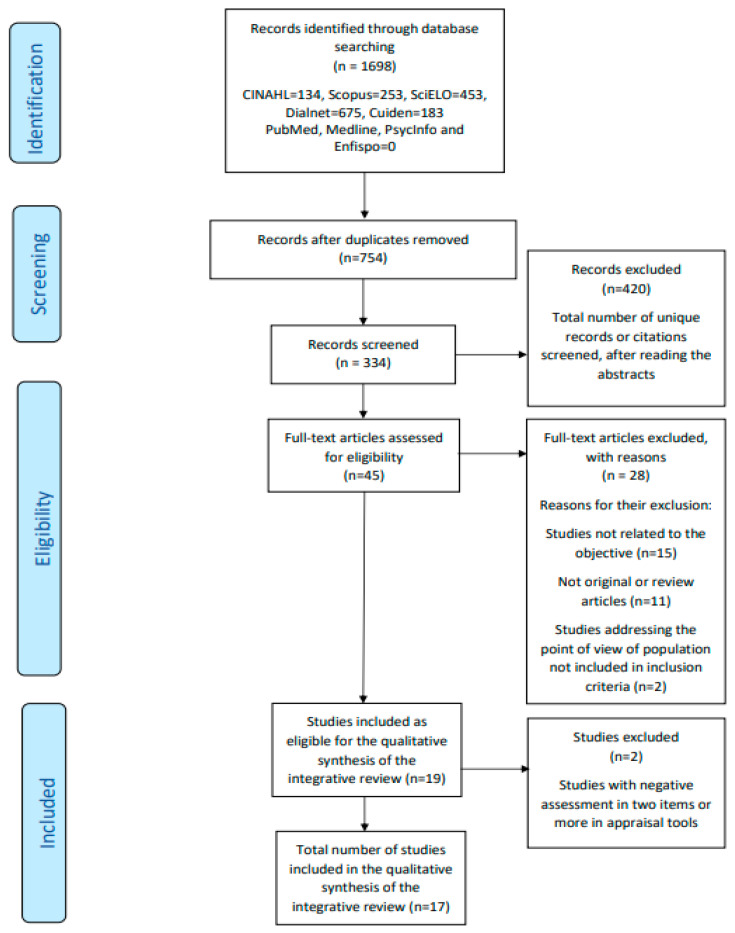
Flow diagram of the integrative review.

Following the described methodology, a total of 17 articles were finally selected. The flow diagram of the information through the different phases of the review, can be seen in Figure 1. 

Selected papers mostly correspond to studies conducted in Spain (n = 7) [1,20,37,38,39,40,41], but studies conducted in Argentina (n = 2) [42,43], Iran (n = 2) [44,45], Egypt (n = 2) [46,47] were included and also from the United Kingdom (n = 1) [9], Venezuela (n = 1) [48], Turkey (n = 1) [49] and Australia (n = 1) [7]. The vast majority of the articles (n = 10) showed the results of quantitative descriptive studies through surveys of different population groups (professionals, nursing students and general population) [7,20,37,38,43,44,46,47,48,49] and some of them (n = 5) were qualitative studies through key informants (professionals, nursing students and/or patients) [1,9,38,42,45]. A literature review [40] and a mixed study that carried out a review of the literature combining a qualitative analysis of the press were included as well [41].

In this way, main results of the review are shown below, collected in Table 1, including the authors, country and year of publication; general objective, methodology and main results.

## 4. Discussion

In the literature reviewed, the characteristic of humanization as an indispensable and inherent element predominates, vocation being the central nucleus in the nurses’ image, considering it as a requirement of the profession [1,37,43]. In this way, the value attributed to the training of the Nursing professionals derives more from their interpersonal skills than from their technical or critical thinking skills. In other words, greater importance is attributed to the fact that characteristics such as kindness and closeness are present in the Nursing personnel than to nurses having undergone good training at the scientific level [1,37,49]. 

Another aspect to be noted is the duality between the Nursing students’ self-image and the external image of the Nursing Profession in a way that it does not coincide with the one held by the Nursing professionals themselves. Society has incorrect information about the functions performed by the Nursing professionals, which is based on myths and stereotypes that exert a negative influence on the image of the Nursing discipline, which, among other things, favors that part of the population follows the Nursing guidelines only after contrasting them with the medical professional or, as will be mentioned later, that the number of students choosing Nursing for their university studies is reduced [9,37,45,48]. 

Given the lack of knowledge among the population regarding the competences of Nursing, its professionals are frequently mistaken for the rest of the health personnel [1,20,40]. In addition, the ATS (Technical Health Assistant) and nurse practitioner denominations persist to a certain extent to refer to the Nursing professionals, thus reinforcing an erroneous image of the profession [1,37]. The same happens with the terms “parastar” and “nurse”, which are used in Iran and in English-speaking countries, respectively, to designate any person that is in charge of providing any kind of care, either in a hospital or at homes (Nursing assistants, people responsible for the home care of children or older adults…). Consequently, by using these terms, reference is made both to the Nursing professionals and to individuals with limited or no training. Therefore, using such terms to refer to Nursing lacks professionalism and exerts a negative influence on the public image [45]. Most of the population knows that Nursing is a university course, but they rank it behind Medicine regarding its social importance [20,37].

Among the competences attributed to the Nursing Profession by part of the population, helping the medical professionals stand out in the first place. They mainly attribute female and male nurses’ activities targeted at providing medical treatments. They also define nurses as those people who are responsible for assisting the patients, without making any reference to the act of caring in most of the cases, this act being understood as the essence of the Nursing Profession. On the other hand, society is unaware of the independent or autonomous functions of the Nursing professionals [1,20,49].

Regarding the view of the Nursing Profession by the rest of the health personnel, it is to be noted that the Nursing and Medical professionals show hierarchical and vertical relationships; in addition, the latter deny the autonomy and specificity of Nursing, since those who practice the medical profession assert that the function of the Nursing professionals is to follow their indications. Consequently, there is tension in the definition of the role between the one supposed by the biomedical model and that prescribed by Nursing. Conversely, the relationships are horizontal with the rest of the health professionals, attributing more recognition and value to the Nursing work [42,43]. 

On the other hand, regarding the Nursing students, in a study conducted in Spain by Albar and Sivianes-Fernández [39], the students attending first year acknowledge the functions of the Nursing professionals in health recovery through care and assistance, as well as by performing techniques such as measuring blood pressure. However, they are unaware of the autonomous role of the Nursing Profession. Based on this, it can be asserted that, at the beginning, the students have an image of the profession that is similar to the one present in the general population, for not having had direct contact with the performance of the Nursing competences. Regarding the students attending fourth year, they highlight health promotion and disease prevention as functions of Nursing, in addition to health recovery. Fourth-year students also make reference to the Nursing professionals and to their competences through assistance, research, teaching, management, health care and promotion, and disease prevention, highlighting the following characteristics: empathy, scientific knowledge, autonomy, and the importance of knowing how to work as a team. On the other hand, similar results are observed in a study conducted in Venezuela by Restrepo, Roberti and Zambrano [48]. Consequently, the students attending the first semester indicated the provision of specific and direct care to the users focusing only on the disease process as the most important function of the Nursing Profession. However, the students attending the tenth semester pointed out that the most important functions of the nurses are to prevent diseases in school centers, industries, child circles and the community, thus acknowledging the important work of the Nursing Profession as an educator in health, beyond pathological processes and the hospital setting. In view of all of the above, a positive evolution of the Nursing students’ self-perception regarding their image throughout the educational process has been discovered, since at the beginning, they start their studies with a perception that is similar to that of the public opinion of the profession and they gradually get to know the discipline better, constructing a self-image that is more in accordance with its reality [39,45,46,48].

Male and female Nursing professionals show a common self-image, which, as already mentioned, is centered on care, which must be comprehensive and holistic [42]. Consequently, the terms used by the very individuals who exercise the Nursing Profession to refer to the benefits of being a nurse are diversity, privilege and compliance. Nursing professionals feel privileged of being present in all the important moments of people’ lives and consider themselves indispensable in the health system where they work since, without their performance, it would be impossible for that system to progress. The Nursing collective also feels privileged of being those who remain longest with the patients and, in addition, they are the first professionals who come into contact with the users when they arrive at the health institution. The Nursing professionals consider it negative that, to construct the social image of the profession, their own Nursing functions are related to unpleasant situations or to stress and believe that, among other factors, the conception that still persists in society about the Nursing Profession is a consequence of its image throughout history, as well as of the behavior of the Nursing professionals themselves since, in numerous occasions, they minimize their importance in the system and do not intervene to improve their image [9,44,45,47].

The social image of Nursing is blurred and certain gender stereotypes about the Nursing Profession and its functions still persist [1,38]. However, predominantly in all the groups, Nursing is considered as a profession that can be practiced both by men and by women [44,45,48]. Therefore, a less stereotyped perception than some years ago is observed [39], even with one of the studies found making reference to certain masculinization accepted by the population, qualifying the care provided by male nurses as excellent [20]. All of the above contrasts another study found which addresses the image of Nursing in the population from the perspective of Iranian male nurses, since the study makes a reference to a feminized Nursing Profession, which, alongside with the social conception of Nursing as subordinated to the medical figure, results in a minimal percentage of men working in the profession. However, it is worth noting that, although the Iranian male nurses advocate that the Nursing Profession can be practiced in an adequate manner both by men and women, they intend to solve the gender issue by performing their functions in the scopes of management, armed forces, and in the emergency and intensive care services, because they believe that male Nursing professionals are more appropriate in services where more speed, high technology and less contact are required. On the other hand, they consider that female nurses are more qualified to develop their competences in the areas of pediatrics, maternity and community health [45]. 

In its turn, the image of Nursing in the most renowned communication media frequently reflects stereotypes that are degrading for the profession; for that reason, the Nursing professionals consider it a factor that exerts a negative influence on the construction of the social image of the Nursing Profession [40]. Consequently, it is spoken of a distorted image of Nursing, since the Nursing collective is shown in a negative way because its skills are not reflected, whereas the medical professionals are portrayed in a positive manner [7,9,45,46,47]. 

In summary and in order to respond to the proposed objectives, the reviewed bibliography on the image of nursing has contributed, as main findings, several approaches:

1. Humanization and vocation as a requirement of Nursing Profession, since the reviewed bibliography largely emphasizes this aspect.
2. The duality between the internal and external image of the Nursing Profession. This includes the analysis that the revised bibliography carries out of the different images that are perceived of the Nursing Profession from different perspectives (population, health professionals, nursing students...) and also the analysis of the self-image of the professionals of nursing.
3. The strong component related to gender stereotypes in the Nursing Profession, very present in the findings of the reviewed bibliography.
4. The Nursing Profession in the media, which sometimes offers a distorted image, generating an erroneous vision of nursing that is highly accentuated in the technical component of the profession and in stereotypes.

In any case, some limitations in the review that could influence results that do not have a high level of validity should be highlighted. In fact, the external validity was not measured, because the setting and nature of the studies differed, for instance, designs (quantitative descriptive designs, qualitative designs, even literature reviews), cultural contexts, characteristics of participants, and so on; sometimes with difficulties to compare their findings, hence undermining external validity.

A search was not performed on grey literature pages or secondary sources. This can lead to increased publication bias, because this sort of analysis about social image could even be expected to be published in this sort of literature. It should also be noted that in the selection criteria, in order to facilitate the analysis, some limitations have been established, such as limitations by language or by dates, which may have led to the loss of interesting information for the analysis and conclusions of this review.

## 5. Conclusions

In a general way, it can be asserted that Nursing is a deeply unknown and invisible profession, because society still does not acknowledge its competence, autonomy and independence. Consequently, certain duality predominates between the self-image of those who exercise the Nursing Profession and its external image. For that reason, it is considered fundamental that the Nursing professionals reflect on what they are and on what they want to be and to convey. In addition, it is crucial that they proclaim their competences so that the population is aware of all that they can offer because, otherwise, society will hardly identify their essence. 

On the other hand, it can be asserted that there is certain inconsistency between the high levels of training attained, and the real recognition and status of the profession. Consequently, so that the image of the Nursing Profession is more in accordance with reality, professional progress must continue. To such end, encouraging the strengthening of autonomy, identity, status, the appropriation of the knowledge corpus, and the struggle to defend unionization must be the urgent commitment to achieve greater social recognition. Consequently, it is important to devise different proposals aiming at strengthening all the dimensions that need improvement so as to enhance positioning and to allow that the image of Nursing in society is adjusted to reality.

Therefore, it is fundamental that the Nursing professionals reflect on what they have conquered and on the situation in which they are, so as to adequately define what they are and what they want to be, projecting it to society to give visibility to their essence. 

## Figures and Tables

**Table 1:** Results of the review.

Authors, Country (year)	General Objective	Method	Sample	Main Results
Restrepo, L., Roberti, JA. and Zambrano, N. Venezuela. (2015) [48]	To determine the perception of the social image of the Nursing Profession in Nursing students.	Cross-sectional and descriptive study. Structured questionnaire with 10 items.	Sample of 143 Venezuelan Nursing students attending the first, seventh and tenth semesters.	The students attending the first semester state that the most important function of Nursing is that of providing specific and direct care to the users based on their needs and problems. The students attending the tenth semester consider that the most important function of female and male nurses is to prevent diseases in school centers, industries, child circles, and the community. The social image of the profession does not correspond to the one they have.
Samaniego, C., Cárcamo, S. and Frankel, D. Argentina. (2011) [42]	To know the current social image of Nursing in the work context of different public and private hospitals.	Qualitative study.	Sample of 55 individuals, interviewed with open questions. (21 Nursing professionals, 21 professionals from other health areas, and 13 users of the Argentinian health system).	Hierarchical and vertical relationships appear with the physicians; in addition, these professionals deny the autonomy and specificity of Nursing. Tension in the definition of the role between the one supposed by the biomedical model and that prescribed by Nursing. The relationships are horizontal with the rest of the health professionals, greater recognition and appreciation being given to the Nursing Profession. They agree in that comprehensive and holistic care is the essence of the Nursing Profession.
De Nova, L. and Vargas-Machuca, FA. Spain. (2011) [1]	To explore the social image that the patients have of Nursing, so as to deepen on their perception of the nurses and of the functions they perform.	Qualitative study. Semi-structured interviews	Targeted at 15 patients in the Virgen Macarena University Hospital of Seville, Spain, belonging to the Andalusian public health system.	The nurses’ participation is more valued in view of their interpersonal skills than due to their technical or critical thinking skills. Blurred social image, in which gender stereotypes persist. Nurses are mistaken for other health professionals and the work they perform is ignored. The Technical Health Assistant (Asistente Técnico Sanitario, ATS) denomination persists to refer to the Nursing professionals. Most of the interviewees answered that the Nursing duty is “to assist the patients”.
Morris-Thompson, T., Shepherd, J., Plata, R. and Marks-Maran, D. United Kingdom. (2011) [9]	To explore the image that the Nursing collective has about its profession and the population’s perception of Nursing.	Qualitative study. It is conducted through focus groups and interviews.	Sample of 159 individuals: nurses who work in London, United Kingdom, as well as with the participation of the population in general.	The population is misinformed about the functions performed by the Nursing professionals. The image they have is based on myths and stereotypes. The self-image of Nursing can be described with the following words: diversity, privilege and compliance, since they are the main terms with which they define the benefits of being a nurse. Invisibility or distorted projection of Nursing in the communication media as a barrier to perceive a real image of the profession. The perception held by the Nursing professionals does not coincide with the one present in society.
Varaei, S., Vaismoradi, M., Jasper, M. and Faghihzadeh, S. Iran. (2012) [44]	To describe the nurses’ perception with respect to the factors influencing their public image.	Descriptive study conducted with a questionnaire.	Participation of 220 Iranian female and male nurses.	They consider it positive that the population attributes Nursing the function of providing care or comfort during a disease process. They consider it negative that the Nursing work is associated with unpleasant situations or stress. Nursing as a profession targeted both at men and women.
Samaniego, C. and Cárcamo, S. Argentina. (2013) [43]	To describe the image that the nurses, the medical collective, and the rest of the health professionals have about the Nursing professionals.	Descriptive study. Questionnaires with multiple-choice closed questions were used.	Sample of 308 participants (100 female and male nurses, 103 medical professionals, and 105 professionals from other areas) from Buenos Aires, Argentina.	Overload and conflicts in the working group are more frequent among the Nursing personnel. Discrepancies between what the nurses consider as their ‘specific functions’ and the opinion of the other health professionals. For the Nursing personnel and other health professionals, the nurses’ functions are centered on care. However, the physicians claim that the function of Nursing is to follow their indications. There is tension between the role prescribed by the medical model and the one defined by Nursing.
Weaver, R., Salamonson, Y., Koch, J. and Jackson, D. Australia. (2013) [7]	To explore the perception held by the Nursing students regarding how the profession is represented in health-related TV programs.	Quantitative study.	Sample of 484 Nursing students from New South Wales, Australia.	The students are aware that television can be a negative influence for the image of Nursing, but they also acknowledge some of its educational values. They assert that the medical professionals are shown in a positive manner, whereas the Nursing collective is portrayed negatively, because their skills are not shown.
El Rahman, R.M.A. and Abou Shousha, A.A.E.F. Egypt. (2013) [46]	To investigate the perception held by the Nursing students from Damanhour, Egypt, about the public image of the profession.	A quantitative-descriptive study through a questionnaire.	Participants were 904 students attending each of the four undergraduate years and the practice year of the Nursing course	The information obtained about Nursing before starting their training comes from family members and friends in the first place, followed by the communication media. They show an improvement in the perception of the Nursing image after starting their university studies, mainly due to the working conditions, to their family members’ opinions, and to the influence of the faculty.
Valizadeh, L., Zamanzadeh, V., Fooladi, MM., Azadi, A., Negarandeh, R. and Monadi, M. Iran. (2014) [45]	To explore how male nurses perceive the public image of Nursing and their self-perception.	Qualitative-descriptive study. The study will be conducted by means of semi-structured interviews.	The sample consists of 18 male nurses working in the Tabriz Hospital, Iran.	The participants stated that the most important factor that makes it difficult for men wishing to become nurses is the public image of the profession as female and subordinated to the physician. They believe that the public image of the profession is influenced by the nurses’ behavior and by the image of the Nursing Profession throughout history. They assert that the communication media, as well as the gender stereotypes transmitted by the educational system, exert a negative influence on the public image of the profession. It can be asserted that the public image of Nursing does not coincide with the professionals’ self-image.
Keçeci, A., Celik Durmuş, S., Oruç, D. and Öner Kapisiz, O. Turkey. (2014) [49]	To determine the population’s perception regarding the competences of the Nursing Profession.	Descriptive study.	The study population is made up of 458 individuals from the Merket District, Düzce, Turkey	The population thinks that the work of female nurses is mainly to support the physicians. They associate Nursing mainly with activities targeted at providing medical treatments. The independent or autonomous functions of Nursing are not correctly understood by society. They assert that nurses only possess good interpersonal skills and claim that the future Nursing professionals must also be qualified.
Rodríguez, MD., Rodríguez, MM. and Tortosa, V. Spain. (2015) [37]	To know the social image that the population has about the Nursing professionals.	Cross-sectional and descriptive study.	Sample of 119 individuals from Almería (Spain)	Most of the population asserts that the nurses have their own functions and show respect for the collective. Regarding social importance, they rank Nursing behind medicine and teaching. The majority knows that Nursing is a university course. The ATS (Technical Health Assistant) and nurse practitioner denominations remain present and, in some cases, they only follow the Nursing recommendations if they are contrasted with the medical figure.
Muñoz, R. and Consuegra, MD. Spain. (2015) [20]	To identify the social image of Nursing through the non-health population.	Cross-sectional and descriptive study through a self-administered questionnaire.	Sample of 220 non-health individuals from Madrid	The general population prefers that there are Nursing professionals of both genders, and they qualify the care provided by male nurses as excellent. Administration of injectables and helping the physician as main functions. Lack of knowledge about the functions performed by the nurses, but they show respect and admiration for these professionals, obtaining good appreciation. They rank the Nursing collective behind the physicians.
Aranda, M., Castillo-Mayén, MR. and Montes-Berges, B. Spain. (2015) [38]	To analyze if a traditional view of the Nursing profession still exists in relation to the stereotypes and gender roles attributed to male and female Nursing professionals.	Qualitative study.	The participants were 12 patients, non-patients and Nursing students from the province of Jaén, Spain.	The gender stereotypes attributed to male and female nurses present some similarities; therefore, a less stereotyped perception than some years ago is observed. A traditional attribution of stereotypes and gender is thus shown between male and female Nursing professionals, even among the group of Nursing students.
Albar, MJ. and Sivianes-Fernández, M. Spain. (2015) [39]	To identify the perception of the professional identity of Nursing in the students attending the first and fourth years of the undergraduate course.	Descriptive study. Survey with 14 items and two open questions.	Random sampling of 50 Nursing students attending first year and 51 students from fourth year at the University of Seville.	Most of the students attending first year acknowledge the role of Nursing in health recovery, and they are in disagreement with the autonomy of the profession. The students attending fourth year highlight health promotion and disease prevention, in addition to health recovery, as functions of Nursing. They make references to assistance, research, teaching, management, care and health promotion, and disease prevention, highlighting the following characteristics: empathy, scientific knowledge, autonomy, and the importance of knowing how to work as a team.
Baldrich Rodríguez, I., Navarro Revueltas, C. and Lázaro Maeso, A. Spain (2016) [40]	To know what Nursing conveys or communicates to society in Spain, from the 20th to the 21st century.	Bibliographic review.		Stereotypes, preconceptions, medical subordination, unawareness of Nursing competences, and sexist image. Respect and admiration for the profession.
Sánchez Gras, S. Spain. (2017) [41]	To present a critical and thorough analysis of the treatment given by the written press to the profession and to the Nursing professional	Bibliographic review and qualitative-analytical study using the news published in the regional and national written press where Nursing is mentioned as sources.		Secondary role associated with another profession, with no responsibility, autonomy or capacity in decision-making.
Mohamed Abdelrahman, S. Egypt. (2018) [47]	To identify the relationship between the social image of Nursing, self-image, and the Nursing professional’s self-esteem.	Descriptive study. 28-item survey that assesses the social image of Nursing.	320 female nurses who work in the Minia hospital.	Positive correlation between professional self-image and social image. Slightly positive correlation between social image and professional nurses’ self-esteem. Negative correlation between professional self-image and female nurses’ self-esteem.

## References

[1] De Nova de la Mata L., Vargas-Machuca Guerrero F.A. (2011). Percepciones de los pacientes sobre la enfermera y su trabajo. Estudio cualitativo en un hospital de Sevilla. Investigación Y Género, Logros Y Retos. III Congreso Universitario Nacional Investigación Y Género.

[2] Romero-Martín M., Gómez-Salgado J., Robles-Romero J.M., Jiménez-Picón N., Gómez-Urquiza J.L., Ponce-Blandón J.A. (2019). Systematic review of the nature of nursing care described by using the Caring Behaviours Inventory. J. Clin. Nurs..

[3] Romero-Martín M., Ponce-Blandón J.A., Gómez-Salgado J. (2019). El cuidado expresado en los comportamientos enfermeros desde la perspectiva de los profesionales y de los pacientes. Rol. Enferm..

[4] García Bañón A.M., Sainz Otero A., Botella Rodríguez M. (2004). La Enfermería Vista Desde el Género. Index de Enfermería.

[5] Burguete Ramos M.D., Martínez-Riera J.R., Martín González G. (2010). Actitudes de Género y Estereotipos en Enfermería. Cult. Cuid..

[6] Bernalte Martí V. (2014). Minoría de hombres en la profesión de enfermería. Reflexiones sobre su historia, imagen y evolución en España. Enferm. Glob..

[7] Weaver R., Salamonson Y., Koch J., Jackson D. (2013). Nursing on television: Student perceptions of television’s role in public image, recruitment and education. J. Adv. Nurs..

[8] Calvo Calvo M.Á. (2011). Imagen Social de Las Enfermeras Y Estrategias de Comunicación Pública Para Conseguir Una Imagen Positiva. Index Enfermería Scieloes.

[9] Morris-Thompson T., Sherpherd J., Plata R., Marks-Maran D.I. (2011). Diversity, fulfilment and privilege: The image of nursing. J. Nurs. Manag..

[10] Solano López A.L. (2012). Imagen social de la enfermería en Costa Rica y su construcción desde la autoimagen profesional. Enferm. Costa Rica.

[11] Arreciado A. (2014). Identidad Profesional Enfermera Construcción y Desarrollo en los Estudiantes Durante su Formación Universitaria. http://www.tdx.cat/handle/10803/129270%0Ahttp://hdl.handle.net/2445/49181.

[12] Robledo J., Younis J. (1994). El papel de los medios de comunicación en los Procesos de la construcción de la realidad y sus implicaciones en la intervención social. Ponencia Marco, Área de Medios de Comunicación IV Jornadas de Intervención Social.

[13] Gómez-Serrano C., Munar-Olaya C. (2009). Albores de la enfermería profesional en Colombia. Temperamentum.

[14] González Carrillo E., Arras Vota A.M., Moriel Corral L. (2012). La profesionalización en enfermería: Hacia una estrategia de cambio. Tecnociencia.

[15] Zamorano Pabón I.C. (2008). Gestión en equipo de enfermería. Investig. Educ. Enferm..

[16] Errasti-Ibarrondo B., Arantzamendi-Solabarrieta M., Canga-Armayor N. (2012). La Imagen Social de la Enfermería: Una Profesión a Conocer. An. Sist. Sanit. Navar. Scieloes.

[17] Heierle Valero C. (2009). La Imagen de la Enfermera a Través de los Medios de Comunicación de Masas: La Prensa Escrita. Index Enfermería Scieloes.

[18] Remirez Suberbiola J.M., Pereda Arregui E., Delgado Aguilar H., Delgado Aguilar M.J. (2010). Enfermería y futuro: Su evolución, ¿credibilidad?. Asoc. Española Enfermería Urol..

[19] Velázquez A.S., Carmen M., Aliste Á. Título: Invisibilidad Enfermera: Una Realidad a Combatir. La Raíz Los Cuid Enfermeros 2014. www.fabulacongress.es/certamenraquel/.../InvisibilidadEnfermera.pdf.

[20] Muñoz Cruz R., Consuegra Alférez M.D. (2015). Imagen social de la enfermería en una población no sanitaria de la ciudad de Madrid. Nuberos Científca.

[21] Santa Clotilde Jiménez E., Casado del Olmo M., Fernández Araque A.M. (2006). Opinión de los usuarios sobre la profesión y el trabajo desarrollado por los profesionales enfermeros. Bibl. Lascasas..

[22] Guerra-Martín M.D., Cano-Orihuela A., Martos-García R., Ponce-Blandón J.A. (2021). Translation and First Pilot Validation Study of the “Undergraduate Nursing Student Academic Satisfaction Scale” Questionnaire to the Spanish Context. Int. J. Environ. Res. Public Health.

[23] Romero-Martín M., Gómez-Salgado J., de la Fuente-Ginés M., Macías-Seda J., García-Díaz A., Ponce-Blandón J.A. (2019). Assessment of reliability and validity of the Spanish version of the Nursing Students’ Perception of Instructor Caring (S-NSPIC). PLoS ONE.

[24] Rezaei-Adaryani M., Salsali M., Mohammadi E. (2012). Nursing image: An evolutionary concept analysis. Contemp. Nurs..

[25] García-Carpintero Blas E. (2007). Reflexión del papel de la enfermería a lo largo de la historia. Enferm. Glob. Rev. Electrónica Semest. Enferm..

[26] Organización Mundial de la Salud (2005). Reglamento Sanitario Internacional 2005.

[27] Vilchez Barboza V., Sanhueza Alvarado O. (2011). Enfermería: Una disciplina social. Enferm. Costa Rica.

[28] Willis D.G., Grace P.J., Roy C. (2008). A Central Unifying Focus for the Discipline: Facilitating Humanization, Meaning, Choice, Quality of Life, and Healing in Living and Dying. Adv. Nurs. Sci..

[29] Martínez Díaz J.D., Ortega Chacón V., Muñoz Ronda F.J. (2016). Design of clinical questions in evidence-based practice. Formulation models. Enferm. Glob..

[30] Coughlan M., Ryan F., Cronin P. (2013). Doing a Literature Review in Nursing, Health and Social Care.

[31] Whittemore R., Chao A., Jang M., Minges K.E., Park C. (2014). Methods for knowledge synthesis: An overview. Hear Lung J. Cardiopulm. Acute Care.

[32] Page M.J., McKenzie J.E., Bossuyt P.M., Boutron I., Hoffmann T.C., Mulrow C.D., Shamseer L., Tetzlaff J.M., Akl E.A., Brennan S.E. (2021). The PRISMA 2020 statement: An updated guideline for reporting systematic reviews. PLoS Med..

[33] Porritt K., Gomersall J., Lockwood C. (2014). JBI’s Systematic Reviews: Study selection and critical appraisal. Am. J. Nurs..

[34] Von Elm E., Altman D.G., Egger M., Pocock S.J., Gøtzsche P.C., Vandenbroucke J.P., STROBE Initiative (2007). The Strengthening the Reporting of Observational Studies in Epidemiology (STROBE) statement: Guidelines for reporting observational studies. Lancet.

[35] O’Brien B.C., Harris I.B., Beckman T.J., Reed D.A., Cook D.A. (2014). Standards for reporting qualitative research: A synthesis of recommendations. Acad. Med..

[36] Tricco A.C., Tetzlaff J., Moher D. (2011). The art and science of knowledge synthesis. J. Clin. Epidemiol..

[37] Rodríguez Porcel M.D., Rodríguez M.D.M., Tortosa Salazar V. (2015). ¿Cómo nos ven los usuarios a los profesionales de enfermería?. Imagen. Soc. Rev. Paraninfo. Digit..

[38] Aranda M., Del Castillo-Mayén M.R., Montes-Berges B. (2015). ¿Ha cambiado la percepción sobre los y las enfermeras? atribución de estereotipos y roles de género? [Has the traditional social perception on nurses changed? Attribution of stereotypes and gender roles?]. Acción Psicológica.

[39] Albar M.J., Sivianes-Fernández M. (2016). Percepción de la identidad profesional de la enfermería en el alumnado del grado. Enferm. Clin..

[40] Baldrich-Rodríguez I., Navarro-Revueltas C., Lázaro-Maeso Á. (2016). Imagen de la enfermería en la sociedad española y medios de comunicación = Nursing image in the Spanish society and media. Rev. Española Comun. En Salud..

[41] Sánchez-Gras S. (2017). Imagen de la enfermería a través de la prensa escrita ¿necesitamos visibilizar los cuidados enfermeros?. Cult. Los. Cuid..

[42] Samaniego C., Frakel D. (2011). La imagen profesional de enfermería en su contexto de trabajo. Hologramática.

[43] Samaniego V.C., Cárcamo S. (2013). The nursing imagen and professional identity. The future of a construction. Invest Educ. Enferm..

[44] Varaei S., Vaismoradi M., Jasper M., Faghihzadeh S. (2012). Iranian nurses self-perception–factors influencing nursing image. J. Nurs. Manag..

[45] Valizadeh L., Zamanzadeh V., Fooladi M.M., Azadi A., Negarandeh R., Monadi M. (2014). The image of nursing, as perceived by Iranian male nurses. Nurs. Health Sci..

[46] El Rahman R.M.A., Shousha A.A.E.F.A. (2013). Perceptions of the Public Image of Nursing among Baccalaureate Nursing Students. Life Sci. J..

[47] Abdelrahman S. (2018). Relationship among public nursing image, self-image, and self-esteem of nurses. Nurs. Health Sci..

[48] Restrepo L., Roberti J.A., Zambrano N.S. (2015). Percepción de la imagen social de enfermería entre los estudiantes del Programa de Enfermería Decanato de Medicina UCLA I lapso 2005. Revista EDUCARE UPEL-IPB Segunda Nueva Etapa.

[49] Keçeci A., Çelik Durmuş S., Oruç D., Öner Kapisiz Ö. (2014). The Society’s View of Nursing in Turkey. Hosp. Top..

